# Overexpression of transient receptor potential mucolipin-2 ion channels in gliomas: role in tumor growth and progression

**DOI:** 10.18632/oncotarget.9661

**Published:** 2016-05-27

**Authors:** Maria Beatrice Morelli, Massimo Nabissi, Consuelo Amantini, Daniele Tomassoni, Francesco Rossi, Claudio Cardinali, Matteo Santoni, Antonietta Arcella, Maria Antonietta Oliva, Angela Santoni, Carlo Polidori, Maria Paola Mariani, Giorgio Santoni

**Affiliations:** ^1^ School of Pharmacy, University of Camerino, Camerino, Italy; ^2^ Department of Molecular Medicine, Sapienza University, Rome, Italy; ^3^ School of Biosciences and Veterinary Medicine, University of Camerino, Camerino, Italy; ^4^ Department of Medical Oncology, Polytechnic University of Marche, Ancona, Italy; ^5^ I.N.M. Neuromed, Pozzilli, Isernia, Italy; ^6^ Anatomo-Pathology Operative Unit, AV3 Macerata, Italy

**Keywords:** TRP channels, mucolipin-2, gliomas, proliferation, apoptosis

## Abstract

The Transient Receptor Potential (TRP) superfamily consists of cation-selective and non-selective ion channels playing an important role both in sensory physiology and in physiopathology in several complex diseases including cancers. Among TRP family, the mucolipin (TRPML1, −2, and −3) channels represent a distinct subfamily of endosome/lysosome Ca^2+^ channel proteins. Loss-of-function mutations in human TRPML-1 gene cause a neurodegenerative disease, Mucolipidosis Type IV, whereas at present no pathology has been associated to human TRPML-2 channels.

Herein we found that human TRPML-2 is expressed both in normal astrocytes and neural stem/progenitor cells. By quantitative RT-PCR, western blot, cytofluorimetric and immunohistochemistry analysis we also demonstrated that TRPML-2 mRNA and protein are expressed at different levels in glioma tissues and high-grade glioma cell lines of astrocytic origin. TRPML-2 mRNA and protein levels increased with the pathological grade, starting from pylocitic astrocytoma (grade I) to glioblastoma (grade IV). Moreover, by RNA interference, we demonstrated a role played by TRPML-2 in survival and proliferation of glioma cell lines. In fact, knock-down of TRPML-2 inhibited the viability, altered the cell cycle, reduced the proliferation and induced apoptotic cell death in glioma cell lines. The DNA damage and apoptosis induced by TRPML-2 loss increased Ser139 H2AX phosphorylation and induced caspase-3 activation; furthermore, knock-down of TRPML-2 in T98 and U251 glioma cell lines completely abrogated Akt and Erk1/2 phosphorylation, as compared to untreated cells.

Overall, the high TRPML-2 expression in glioma cells resulted in increased survival and proliferation signaling, suggesting a pro-tumorigenic role played by TRPML-2 in glioma progression.

## INTRODUCTION

Malignant gliomas are the most common type of primary brain tumors [1]. Diffuse gliomas are classified histologically according to the hypothesized line of differentiation as astrocytomas, oligodendrogliomas and oligoastrocytomas. Astrocytomas are pathologically graded in accordance with the World Health Organization as low grade astrocytoma (grade II), anaplastic astrocytoma (grade III) or high grade glioblastoma multiforme (GBM, grade IV). GBM is the most aggressive and prevalent type of glioma, accounting for at least 80% of malignant gliomas [1, 2]. It is one of the most lethal forms of cancer in human, with a median overall survival of 12-18 months. Although new treatments have been developed on the basis of new knowledge about the molecular nature of glioma disease, surgery in association with radiation therapy and chemotherapy remains the standard of care.

The Transient Receptor Potential (TRP) superfamily consists of cation-selective channels that have roles in sensory physiology such as thermo- and osmo-sensation and in several complex diseases such as cardiovascular and neuronal diseases, and cancer [3]. Among the TRP family, mucolipins (TRPML) represent a distinct subfamily of endosome/lysosome Ca^2+^ channel proteins [4]. The TRPML channels are 6 transmembrane-spanning proteins that consist of cytosolic N- and C-termini, and a pore-loop domain between S5 and S6. In mammals, there are three TRPML proteins (TRPML-1-3, also called MCOLN1-3) [5]. Human TRPML-1 mRNA is expressed in several tissues with the highest levels in the brain, kidney, spleen, liver and heart [6]; by contrast, the expression of TRPML-2 and TRPML-3 mRNAs in humans is more restricted [7], with TRPML-3 mainly detected in the thymus, kidney, eye, skin and olfactory/vomeronasal sensory neurons [8] and TRPML-2 in lung, stomach, colon, mammary gland [9, 10]. Loss or function mutations in the human TRPML-1 gene cause Mucolipidosis Type IV (ML4), a devastating neurodegenerative disease; gain-of-function mutations of the mouse TRPML-3 gene result in the varitint-waddler (Va) phenotype [6, 11–13]. Recently, a role of TRPML-2 channel in innate immune responses and in the susceptibility to bacteria and virus infections has been demonstrated [14]. Human TRPML-2 channels localize in endosomal and lysosomal compartments and functional activity was reported at the plasma membrane [15]. In addition, TRPML-2 forms homo- and hetero-multimers with TRPML-1 and/or TRPML-3 [16, 17]. An important role has been proposed for TRPML-2 in trafficking and regulation along the clathrin-independent Arf6-associated endocytic pathway [18]. Human TRPML-2 is Ca^2+^-permeable non selective cation channel, which is inhibited by low extracytosolic pH and activated by phosphatidil-inositol 3,5 biphosphate (PI(3,5)P2), a low abundance endolysosome-specific phosphoinositide [19].

Since no data on the expression and functions of TRPML-2 channels in gliomas has been provided so far, the present work evaluated the expression of TRPML-2 channels in astrocytomas and glioblastomas compared to normal brain tissues (NB), astrocytes (NHA) and neural stem/progenitor cells (NS/PC). In addition, by RNA interference, the role of TRPML-2 in controlling cell survival and proliferation was studied in glioma cell lines.

## RESULTS

### Expression of TRPML-2 gene in normal human brain, astrocytes and neural stem/progenitor cells

**Figure 1:**
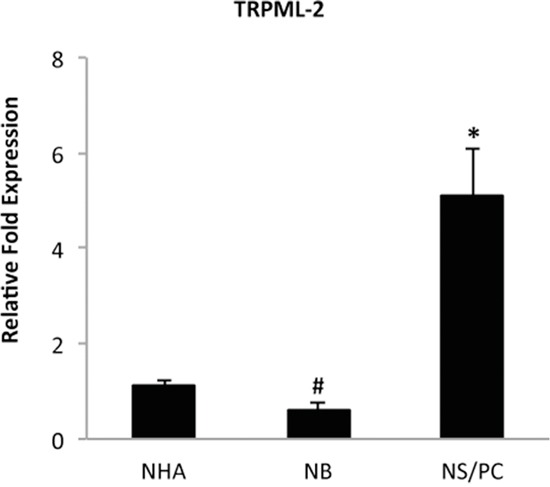
TRPML-2 mRNA expression in normal human brain, astrocytes and neural stem/progenitor cells The relative TRPML-2 mRNA expression in normal human astrocyte (NHA), in human normal brain (NB) and in human neural stem/progenitor (NS/PC) cells was evaluated by qRT-PCR. TRPML-2 mRNA levels were normalized for GAPDH expression and were expressed as relative fold with respect to NHA used as control. Data are expressed as mean ± SD. *p<0.01 NS/PC vs NHA; ^#^p<0.05 NB vs NHA (Anova with Bonferroni's post-test).

We first evaluated by quantitative real time-polymerase chain reaction (qRT-PCR) the expression of TRPML-2 gene in two different normal human brain (NB1 and NB2) samples, astrocyte cell lines (NHA1 and NHA2) and neural stem/progenitor cell lines (NS/PC1 and NS/PC2). We demonstrated that TRPML-2 transcripts were found at higher levels in NS/PCs and NHAs as respect to NB specimens (Figure 1).

### TRPML-2 mRNA expression increases during glioma progression

**Figure 2:**
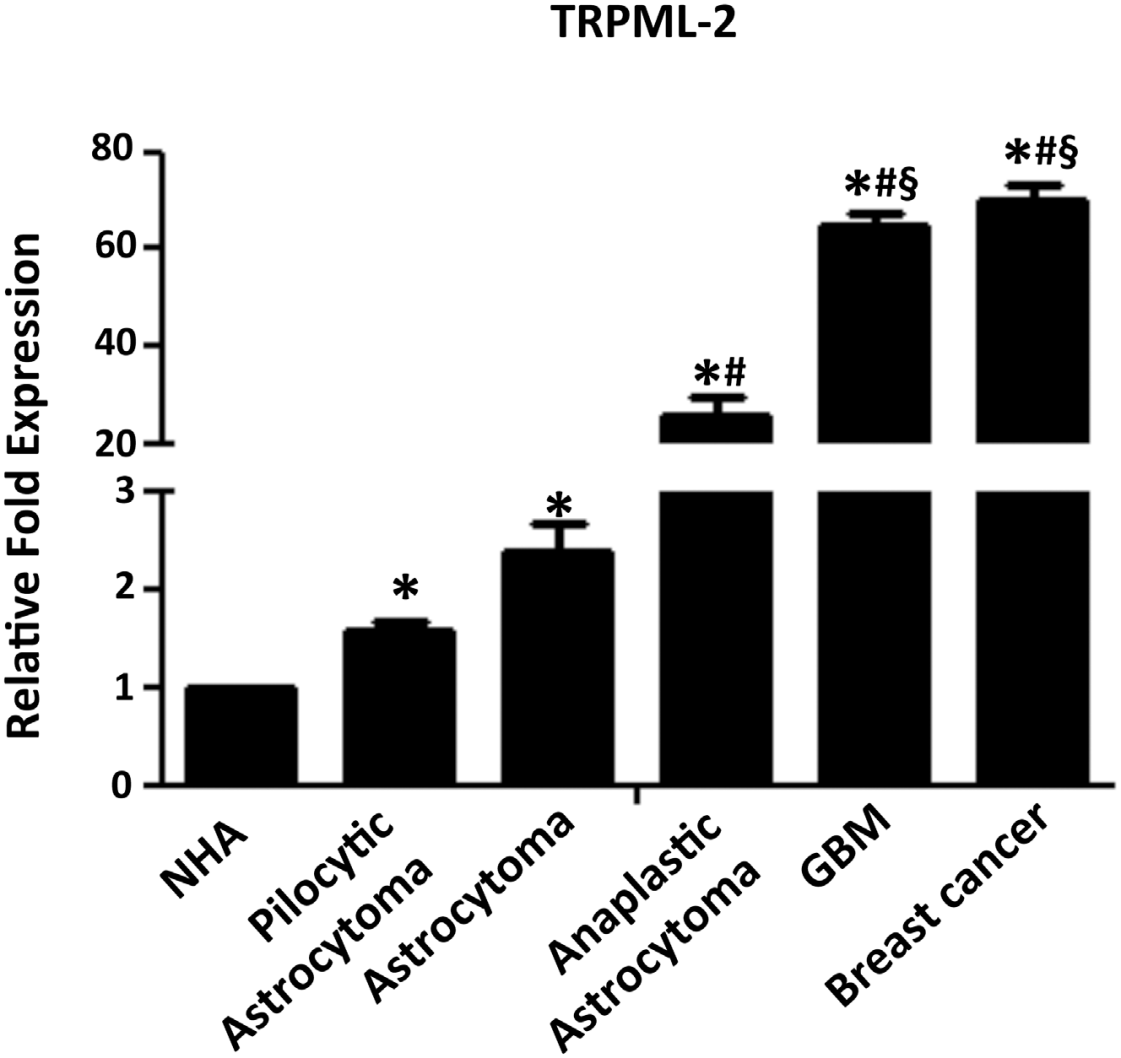
TRPML-2 mRNA expression in glioma tissues The relative TRPML-2 mRNA expression levels in normal human astrocyte (NHA 1,2), glioma tissues (n = 63) at different tumour grades and ER^+^ breast cancers (positive control) were evaluated by qRT-PCR. TRPML-2 mRNA levels were normalized for GAPDH expression and were expressed as relative fold with respect to the mean value of NHA samples, used as control. Data are expressed as mean ± SD. *p<0.05 vs NHA; ^#^p<0.01 vs pilocytic astrocytoma and astrocytoma; ^§^p<0.01 vs anaplastic astrocytoma (Anova with Bonferroni's post-test).

**Figure 3:**
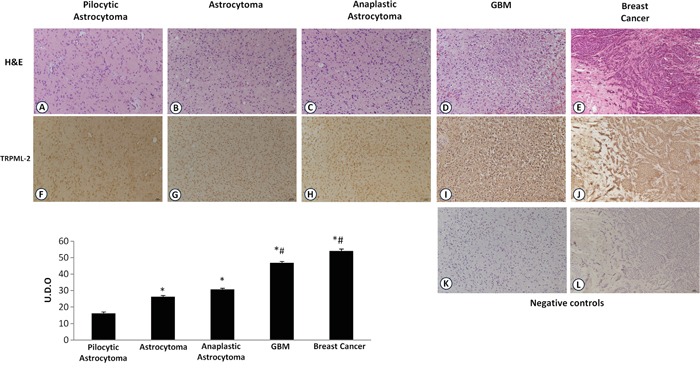
TRPML-2 protein expression in glioma tissues with different pathological grade Immunohistochemical analysis of TRPML-2 protein in human glioma tissues at different pathological stages and ER^+^ breast cancer tissue (positive control). **A-E.** Sections from paraffin-embedded tissues were stained with H&E and **F-J.** Processed for the immunohistochemistry with rabbit anti-human TRPML-2 polyclonal Ab positive TRPML-2 immunoreactivity was present in the plasma membrane and in discrete spots in the cytoplasmic perinuclear area of tumors cells (F-J). **K-L.** Sections incubated with the omission of primary antibody were used as negative control and demonstrated no immunoreactivity images are representative of six slides for each sample. Calibration bar: 25 μm. Bars represent densitometric analysis of glioma tissues. The values, expressed as Unit of Optical Density (U.O.D.) are the mean ± SEM. *p<0.05 vs pilocytic astrocytoma; ; #p<0.05 vs astrocytoma and anaplastic astrocytoma (Anova with Bonferroni's post-test).

The expression of TRPML-2 was also evaluated at mRNA levels in human glioma tissues of both sexes, with different pathological grade (Table 1) and was compared to the mean values of NHA samples (Figure 2). Messenger RNA from estrogen receptor positive (ER+) breast cancer tissues was used as positive control [20]. Starting from low-grade pylocytic astrocytoma, grade I and astrocytomas, grade II towards the more invasive anaplastic astrocytomas, grade III and GBM, grade IV, a progressive up-regulation of TRPML-2 mRNA expression was detected by qRT-PCR compared with NHA samples (Figure 2), with a strong increase (about 45-fold) of TRPML-2 expression in GBM biopsies, compared to pilocytic astrocytomas. The results obtained in glioma tissues prompted us to evaluate the expression of TRPML-2 protein by immunohistochemistry. In agree with qRT-PCR data, astrocytoma, anaplastic astrocytoma and GBM were more immunoreactive, compared to grade I astrocytomas (Figure 3), suggesting an inverse correlation between differentiation status and TRPML-2 protein expression. In addition, no significative difference in TRPML-2 protein expression was found between grade II and III astrocytomas. High TRPML-2 mRNA and protein levels were detected in ER-positive, epidermal growth factor receptor 2 positive (HER2+) breast cancer tissues, used as positive controls (Figure 2 and 3). No reactivity was found in tissue sections used as negative control incubated with the omission of primary Ab (Figure 3).

**Table 1:** Glioma patients characteristics

Patient characteristics	
**N° sample**	n=63
**Sex**	
Men (%)	46
Women (%)	54
**Age, years**	
< 45	n=35
> 45	n=28
**Gliomas Hystology (WHO)**	
Pylocytic Astrocytoma (Grade I)	n=11
Astrocytoma (Grade II)	n=16
Anaplastic Astrocytoma (Grade III)	n=17
GBM (Grade IV)	n=19

Clinicopathological characteristics of the 63 glioma patients analyzed for TRPML-2 mRNA and protein expression.

### TRPML-2 mRNA and protein expression levels in human glioma cell lines

**Figure 4:**
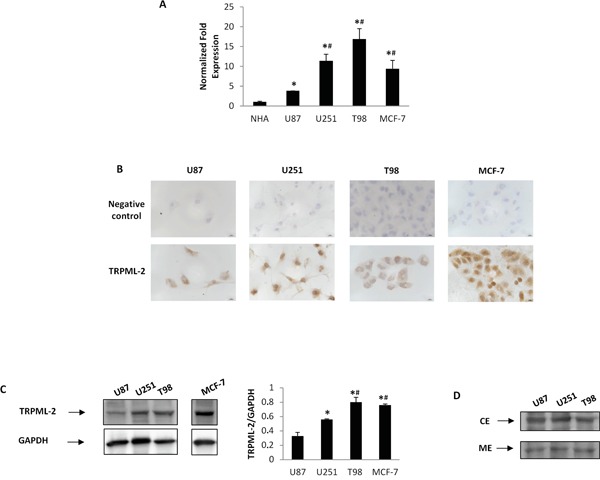
TRPML-2 mRNA and protein expression in high-grade glioma cell lines **A.** The relative TRPML-2 mRNA expression in NHA, U87, U251 and T98 glioma cell lines and in MCF-7 breast cancer cell line used as positive control was evaluated by qRT-PCR. TRPML-2 mRNA levels were normalized for GAPDH expression. Data are expressed as mean ± SD. *p<0.05 vs NHA; ^#^p<0.05 vs U87 (Anova with Bonferroni's post-test). **B.** Immunocytochemical stains for TRPML-2 in glioma cell lines and MCF-7 breast cancer cell line. Cells were formaldehyde-fixed, permeabilized and probed with a rabbit anti-human TRPML-2 polyclonal Ab. Representative images are shown. Calibration bar: 10 μm. **C.** Total lysates were separated on 8% SDS-PAGE and probed with anti-TRPML-2 and anti-GAPDH Abs. TRPML-2 densitometry values were normalized to GAPDH used as loading control. Blots are representative of one of three separate experiments. *p < 0.01 vs U87; ^#^p<0.05 vs U251(Anova with Bonferroni's post-test). **D.** Plasma membrane (ME) and cytoplasm (CE) extracts from U87, U251 and T98 cell lines were immunoblotted with anti-TRPML-2 Ab. Blots are representative of one of three separate experiments.

We further analyzed both at mRNA and protein levels, the TRPML-2 expression in human high-grade T98, U251 and U87 glioma cell lines. By qRT-PCR we observed high expression of TRPML-2 mRNA in T98 and U251 GBM cell lines as well as in MCF-7 cell line, used as positive control, compared to the mean value of NHA samples (Figure 4A). U87 cells showed low TRPML-2 mRNA level compared to T98 and U251 cell lines (Figure 4A). Immunocytochemistry demonstrated a positive immunoreaction for TRPML-2 in all glioma cell lines and in MCF-7 cells (Figure 4B). We further confirmed the protein expression by western blot analysis. In parallel to qRT-PCR data, immunoblots showed that T98 and U251 glioma cell lines displayed a higher TRPML-2 protein level, corresponding to a band of 68 kDa, respect to U87 cells (Figure 4C). Moreover, blots of plasma membrane (ME) and cytoplasm (CE) fraction (Figure 4D) and cytofluorimetric analysis of unpermeabilized and permeabilized glioma cells demonstrated that TRPML-2 localization was both intracytoplasmic and in the plasma membrane, with highest expression in the cytosol (Supplementary Figure 1).

### Silencing of TRPML-2 mRNA inhibits viability and proliferation in glioma cell lines

**Figure 5:**
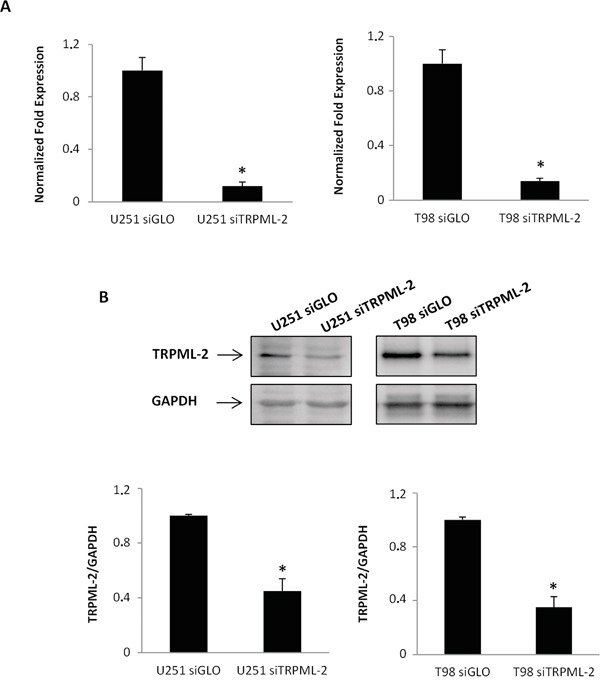
TRPML-2 silencing reduces the TRPML-2 mRNA and protein expression in glioma cell lines **A.** TRPML-2 mRNA levels were evaluated by dxRT–PCR in U251 siTRPML-2 and T98 siTRPML-2 cells after 72 h of transfection. Relative TRPML-2 expression, normalized to GAPDH mRNA levels, was calculated using siGLO as calibrator. Statistical analysis was performed comparing siTRPML-2 with siGLO transfected cells, *p<0.01 (Student's t-test). **B.** Lysates from siTRPML-2 U251 and T98 transfected cells were separated on SDS-PAGE and probed with specific rabbit anti-human TRPML-2 polyclonal Ab. GAPDH protein levels were evaluated as loading control. Relative TRPML-2 expression values were calculated using siGLO as calibrator. Blots are representative of three separate experiments. *p < 0.01 vs siGLO (Student's t-test).

To demonstrate the involvement of TRPML-2 channels in the modulation of glioma cell viability and proliferation, we performed a TRPML-2 gene knock-down experiment in T98 and U251 glioma cell lines. Transfection with siTRPML-2 reduced the TRPML-2 mRNA and protein levels, as evaluated by qRT-PCR (Figure 5A) and western blot analysis in both cell lines (Figure 5B).

**Figure 6:**
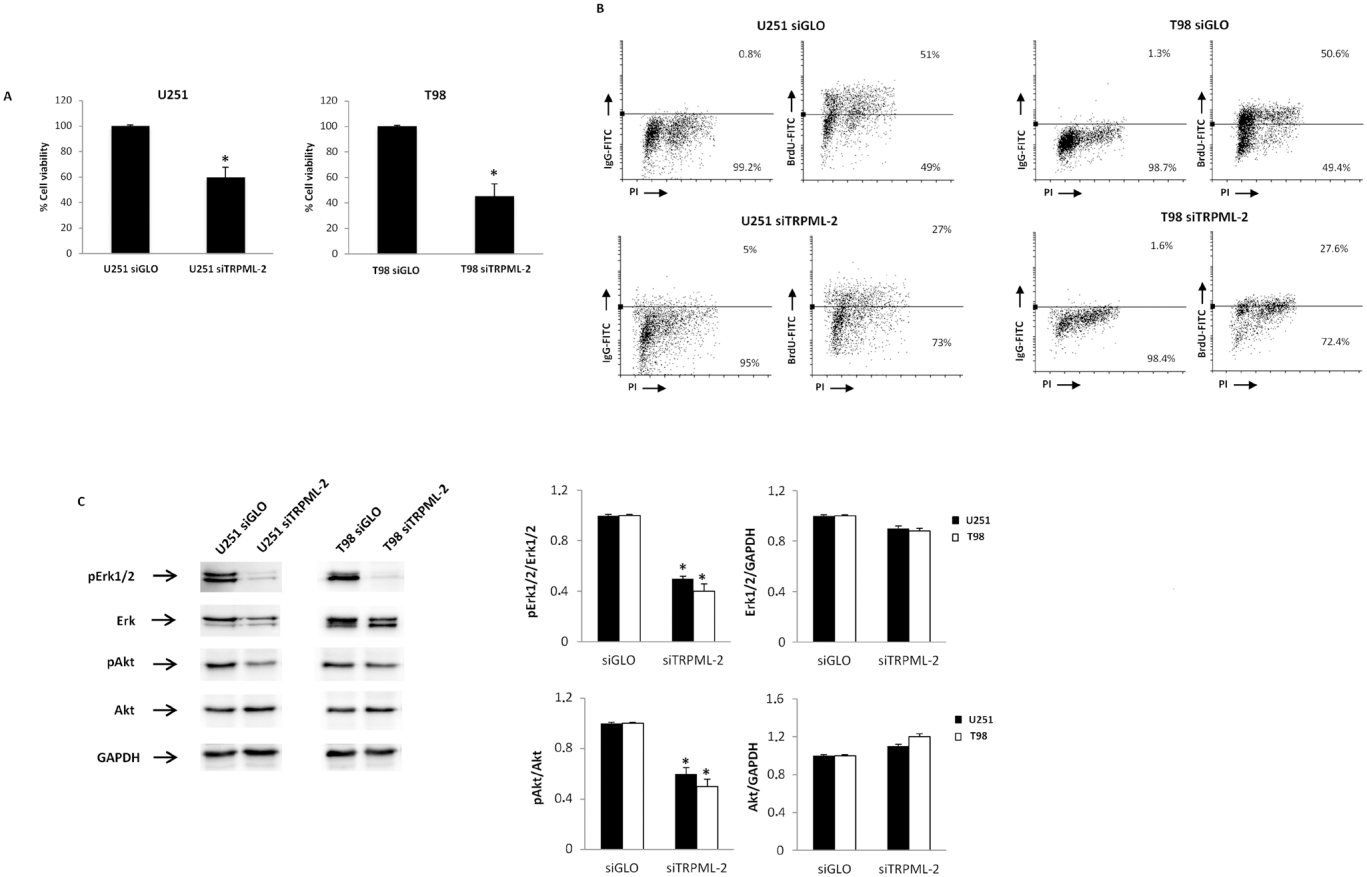
Silencing of TRPML-2 inhibits the viability and proliferation in glioma cell lines **A.** siTRPML-2 U251 and T98 cell viability was determined by MTT assay after 72 h of transfection. Data shown are the mean ± SD of three separate experiments. Statistical analysis was performed comparing siTRPML-2 with siGLO transfected cells, *p<0.001 (Student's t-test). **B.** Cells were transfected for 72 h with siGLO or siTRPML-2 and then pulse labelled with BrdU for 24 h prior to harvesting, and stained with BrdU-FITC conjugate for determination of DNA synthesis, and with PI for determination of total DNA content. BrdU incorporation was determined by quantifying cells positive for BrdU staining in the dot plots. **C.** Lysates from siGLO and siTRPML-2 U251 and T98 cells after 72 h of transfection were separated on SDS-PAGE and probed with specific anti-pErk1/2, anti-ERK, anti-pAkt and anti-Akt Abs. GAPDH protein levels were evaluated as loading control. Blots are representative of three separate experiments. Relative pErk1/2, ERK, pAkt and Akt expression values were calculated using siGLO as calibrator. *p < 0.01 vs siGLO (Student's t-test).

The effect of TRPML-2 knock-down in glioma cells viability and proliferation was evaluated by MTT and BrdU assay, respectively. A reduced viability was evidenced in siTRPML-2 U251 and siTRPML-2 T98 glioma cell lines, respectively, compared with siGLO-transfected cell lines (Figure 6A). Moreover, evaluation of DNA synthesis through BrdU/PI assay showed that TRPML-2 silencing significantly reduced DNA synthesis rate of about 25% in both cell lines after 72 h of transfection (Figure 6B).

The ERK and Akt/PKB pathways regulate the survival and proliferation in gliomas [21]. Therefore, the activation status of Akt/PKB and Erk1/2 proteins was analyzed by western blot analysis. Constitutive Akt and Erk1/2 phosphorylation was observed in T98 and U251 cells. Knock-down of TRPML-2 gene markedly reduced the activation in both cell lines, compared to siGLO cells (Figure 6C).

Overall the reduction in survival and proliferation induced by the loss of TRPML-2 expression is associated with Akt/Erk1/2 pathway inhibition, suggesting a pro-tumorigenic role played by TRPML-2 in glioma progression.

### TRPML-2 knock-down induces DNA damage and cell cycle alteration in glioma cell lines

**Figure 7:**
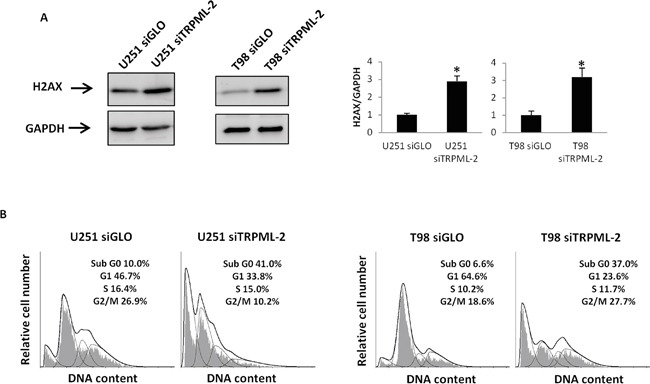
TRPML-2 silencing induces DNA damage and a subG0 cell phase increase in glioma cell lines **A.** Lysates from siGLO and siTRPML-2 U251 and T98 cells after 72 h of transfection were separated on SDS-PAGE and probed with specific anti-H2AX Ab. GAPDH protein levels were evaluated as loading control. Relative H2AX expression values were calculated using siGLO as calibrator. Blots are representative of three separate experiments. Bars represent the densitometric analysis. *p<0.01 (Student's t-test). **B.** Representative cell cycle distribution in U251 siTRPML-2, U251 siGLO, T98 siTRPML-2 and T98 siGLO cells after 72 h of transfection. Cell cycle analysis was evaluated by PI staining and cytofluorimetric analysis. Data expressed as percentage of cells in each phase are representative of one out of three separate experiments.

Inhibition of glioma cell viability and proliferation in siTRPML-2 glioma cells may depend on DNA damage-induced cell cycle arrest. Thus, the effect of TRPML-2 gene silencing on histone γ-H2AX (H2AX) phosphorylation and cell cycle arrest was evaluated by western blot analysis and cell cycle assay. An increased level of Ser-139 γ-H2AX phosphorylation was observed in T98 and U251 siTRPML-2 cells, compared to siGLO cells (Figure 7A). Moreover, knock-down of TRPML-2 increased the percentage of cells arrested at subG0 phase, indicating an elevated percentage of hypodiploid cells with fragmented DNA in U251 and T98 siTRPML-2 cell lines, compared with siGLO cells (Figure 7B).

### The siTRPML-2-mediated DNA damage response triggers apoptosis in glioma cell lines

**Figure 8:**
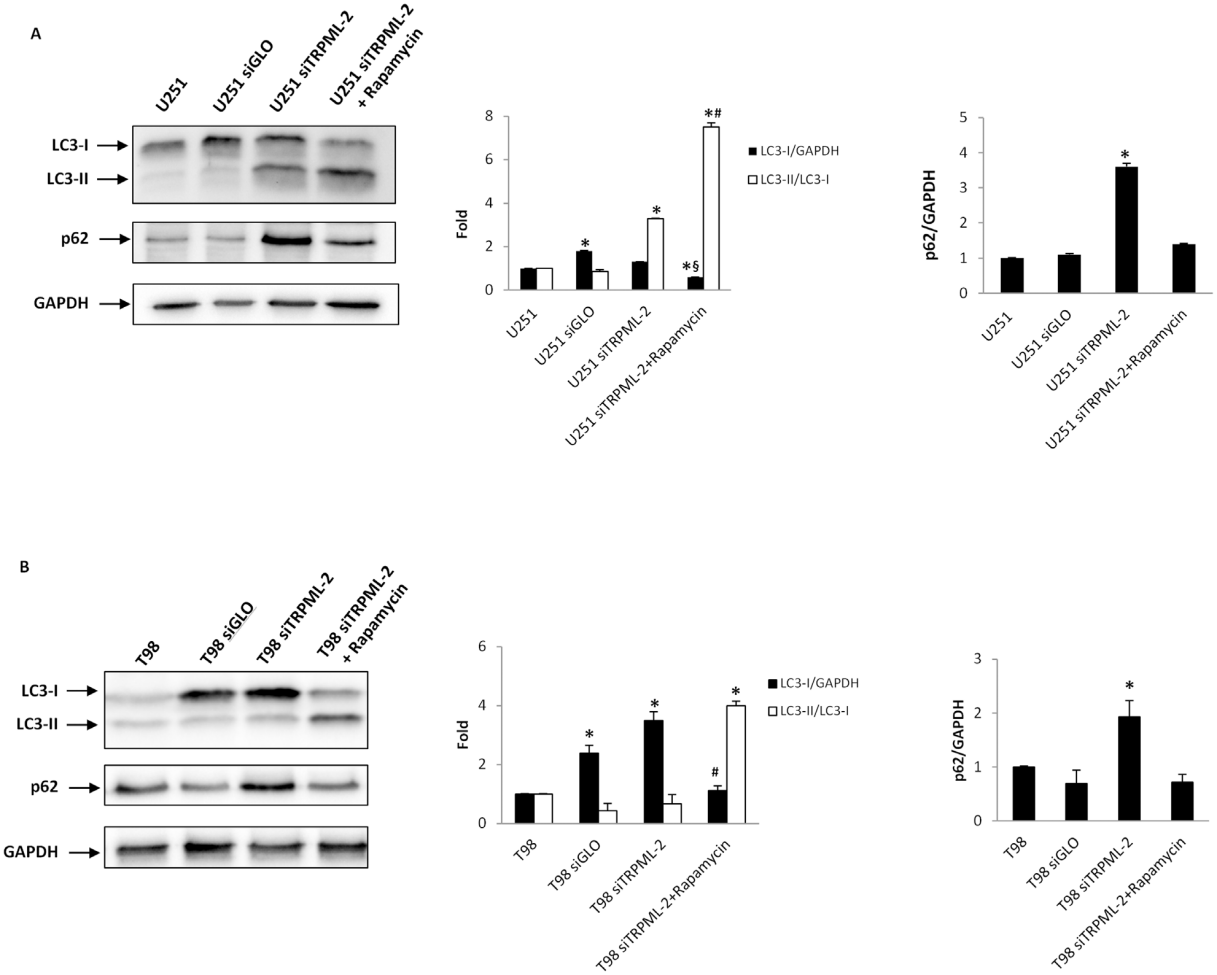
TRPML-2 does not influence autophagic pathway **A.** Lysates from U251 untransfected, siGLO and siTRPML-2, treated or not with rapamycin (50 nM) for 72 h, were separated on 14% SDS-PAGE and probed with anti-LC3, p62 and anti-GAPDH Abs. GAPDH protein levels were evaluated as loading control. Blots are representative of one of three separate experiments. *p<0.01 vs U251; ^#^p<0.01 vs U251 siTRPML-2;^§^p<0.01 vs U251 siGLO or U251 siTRPML-2 (Anova with Bonferroni's post-test). **B.** Lysates from T98 untransfected, siGLO and siTRPML-2, treated or not with rapamycin as above described, were separated on 14% SDS-PAGE and probed with anti-LC3, p62 and anti-GAPDH Abs. GAPDH protein levels were evaluated as loading control. Blots are representative of one of three separate experiments. *p<0.01 vs T98; ^#^p<0.01 vs T98 siGLO or T98 siTRPML-2 (Anova with Bonferroni's post-test).

Autophagy regulates both cell survival and death during stress conditions in different cell types [22]. Therefore, to evaluate the role of TRPML-2 in autophagy, rapamycin, a potent autophagic inducer was used. In particular, U251 and T98 siTRPML-2 cells were treated with rapamycin 50 nM for 72h and modulation of LC3 and p62 proteins was assessed by western blot analysis (Figure 8A and 8B). During autophagy induction, LC3-I isoform is converted into LC3-II to allow the insertion of this protein into autophagosome membrane. Therefore, the amount of LC3-II correlates with the extent of autophagosome formation. Immunoblots revealed that the transfection condition increased the LC3-I levels in both cell lines; moreover, in siTRPML-2 U251 cells increased levels of LC3-II were detected under basal conditions (Figure 8A). When U251 and T98 siTRPML-2 cells were treated with rapamycin to stimulate autophagy, an increased LC3-II and a decreased p62 levels, as respect to basal condition, was observed (Figure 8A, 8B). Overall, these results indicate that TRPML-2 doesn't seem to be involved in the autophagy of glioma cells.

**Figure 9:**
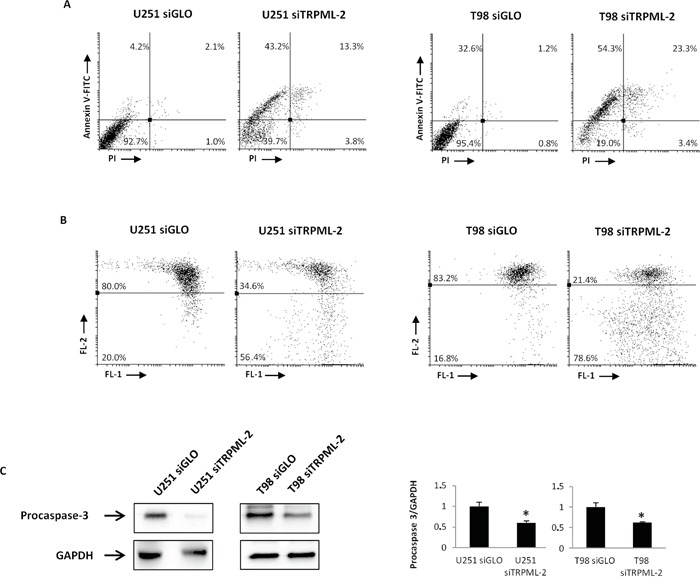
TRPML-2 knock-down induces apoptotic cell death in siTRPML-2 glioma cell lines **A.** Biparametric flow cytometric analysis were performed on U251 siTRPML-2, U251 siGLO, T98 siTRPML-2 and T98 siGLO cells by Annexin V-FITC and PI staining after 72 h of transfection. Cells in the upper left quadrant indicate Annexin V-positive, early apoptotic cells. The cells in the upper right quadrant indicate Annexin V-positive/PI-positive, late apoptotic cells. **B.** The ΔΨ_m_ changes in U251 siTRPML-2, U251 siGLO, T98 siTRPML-2 and T98 siGLO cells after 72h of transfection were evaluated by JC-1 staining and biparametric FL1(green)/FL2(red) flow cytometric analysis. Numbers indicate the percentage of cells showing a drop in ΔΨ_m_-related red fluorescence intensity. Data are representative of one out of three separate experiments. **C.** Lysates from siGLO and siTRPML-2 U251 and T98 cells after 72 h of transfection were separated on SDS-PAGE and probed with specific anti-caspase-3 Ab. GAPDH protein levels were evaluated as loading control. Relative caspase-3 (35 kDa) expression values were calculated using siGLO as calibrator. Blots are representative of three separate experiments. Bars represent the densitometric analysis. *p<0.05 (Student's t-test).

The response to DNA damage results in repair of DNA lesions or caspase cascade activation and apoptosis. Therefore, the triggering of apoptosis in siTRPML-2 glioma cells as well as in siGLO control cells was investigated by Annexin V/PI staining and biparametric cytofluorimetric analysis. Concordantly with the increase of subG0 cell phase populations, the number of early, Annexin V^+^ and late, PI^+^/Annexin V^+^, apoptotic cells was enhanced in siTRPML-2 T98 and U251 cells, as compared to siGLO cells (Figure 9A).

To elucidate the molecular mechanism by which the silencing of TRPML-2 induced apoptotic cell death, the mitochondrial transmembrane potential (ΔΨm) was analyzed in glioma cells. Mitochondrial depolarization was evident after 72 h of transfection in siTRPML-2 cells, as respect to siGLO cells (Figure 9B). In addition, by western blot analysis a reduced level of procaspase-3 protein, as result of caspase-3 activation, was observed in siTRPML-2 T98 and U251 cells, compared to siGLO cells (Figure 9C).

Taken together, loss of TRPML-2 in glioma cell lines induces DNA damage, reduces cell proliferation, and activates caspase-dependent apoptosis.

## DISCUSSION

Changes in TRP channel expression were found in gliomas, and a significant relationship between overexpression of TRP genes and survival of patients has been recently reported [23–25].

Here, we evaluated the expression of TRPML-2 at mRNA and protein levels in glioma tissues with different pathological grades. We observed that TRPML-2 mRNA expression is increased in all glioma specimens analyzed, as compared to NHA. In particular, the expression of TRPML-2 mRNA increased with the pathological grade, from the pylocytic astrocytoma, grade I to the GBM grade IV, with the last showing the highest TRPML-2 mRNA levels. Similarly, we also found that TRPML-2 protein is expressed in all glioma specimens, with the high expression in GBM and low expression in astrocytoma grade I. Concordingly, high level of TRPML-2 mRNA and proteins were observed in high-grade U87 astrocytoma as well as in T98 and U251 GBM cell lines. TRPML-2 protein localizes both in the plasma membrane and in the cytoplasm. In line with our findings, the transcriptional activator of the TRPML-2 gene [26], Paired box 5 (PAX5), also known as B-cell lineage specific activator protein, has been found to be overexpressed in human astrocytoma tissues, and its expression correlates with increasing malignancy and pathological grade [27]. The PAX proteins promote tumor cell survival, proliferation and apoptosis resistance and a reduction in PAX gene expression induces apoptosis in tumor cells [28, 29]. Taken together, these results demonstrate for the first time the expression of TRPML-2 mRNA and proteins in gliomas, suggesting that these channels play an important role and can interfere with gliomas growth.

At present limited data in the physiologic and pathologic role played by TRPML-2 has been provided. So, we evaluated the role of TRPML-2 in glioma cell survival, proliferation and death, by silencing the TRPML-2 gene in T98 and U251 GBM cell lines. We found that siTRPML-2 U251 and T98 cells show a reduced viability, compared to siGLO cells, correlated with a decreased cell proliferation. Furthermore, silencing of TRPML-2 resulted in a strong reduction in G1 phase cell populations, with a marked increase of subG0 DNA fragmentation, a sign of an enhanced cell death. Thus, an increased percentage of Ann V^+^ and PI^+^/Ann V^+^ apoptotic cells was evidenced in siTRPML-2 U251 and T98MG cells, respect to relative controls. Finally, the cellular response to DNA damage induced by TRPML-2 loss, leading to cell cycle arrest involves the γ-H2AX phosphorylation, caspase-3 activation and mitochondria-dependent apoptosis as evaluated by increased Ser-139 γ-H2AX, and reduced ΔΨm and procaspase-3 levels in siTRPML-2 T98 and U251 cells, compared to siGLO cells. In agreement with our results, knock-down of TRPML-2 expression in HEK-293 cells causes severe cell degeneration, characterized by lysosomal inclusions and mitochondrial fragmentation, suggesting that the presence of functional TRPML-2 channel is necessary to ensure cell viability [7]. Finally, since the role for TRPML-1 and −3 channels in autophagy [30, 31], the involvement of TRPML-2 in this pathway was evaluated. In agree with previous findings obtained in TRPML-2 silenced HEK-293 and Hela cells [32], the silencing of TRPML-2 gene in glioma cell lines does not affect the autophagic process.

Changes in the activation and/or expression of TRP calcium channels affect calcium-dependent signalling pathways implicated in tumorigenesis and tumor progression [33]. Increased phosphatidil-inositol-3-kinase/Akt/Protein kinase B (PI3K/Akt/PKB) and extracellular signal-regulated kinase (Erk1/2) pathway activation were found in invasive GBM cells [34–36], and a cross-talk between PI3K/Akt/PKB and Raf-1/MEK/Erk signaling pathways has been reported in GBM cells [37]. In the present study, we found endogenous activation of Akt/PKB and Erk1/2 pathways in T98 and U251 GBM cell lines, that is completely abrogated in siTRPML-2 cells.

The findings of the dependence of TRPML-2-mediated survival and proliferation of glioma cells to the PI3K/Akt pathway may be relevant in the view of a role of phosphoinositides (PIPs) in the regulation of TRPMLs expression and functional activity [19, 38] and on the ability of PI(3,5)P2 to activate TRPML-2 [19]. The PI(3,5)P2 levels are tightly regulated and involved in signaling response to hyperosmotic stress and growth factors in mammalian cells [39, 40]. It has been suggested that different PIP isoforms determine the trafficking and the functional activity of TRPML ion channel between specific cell compartments in the endocytic pathway (endosome/lysosome) and plasma membrane [41]. Thus it is tempting to speculate that tuning of Akt/PKB and Erk1/2 pathways by TRPML-2 in the glioma microenvironment may regulate the survival and proliferation of GBM cells.

Finally, our findings evidenced that the level of TRPML-2 mRNA and proteins was high in neural stem/progenitor cells; since, the glioblastoma stem-like cells, suspected to support the gliomagenesis, derive from neural stem and/or progenitor cells or differentiated cells such as astrocytes or oligodendrocytes [42], the peculiar expression of TRPML-2 channels in neural stem/progenitor cells and astrocytes may be particularly interesting to stimulate additional studies on the expression and functions of these channels in glioma stem-like cells.

The low TRPML-2 expression in normal brain and the progressive overexpression in low vs high grade gliomas may give TRPML-2 channels as predictive and specific biomarker in brain tumors of astrocyte origin [43] and may point to novel approaches in glioma therapy. Understanding the role of TRPML-2 in the regulation of survival and proliferative pathways could shed light on the mechanisms of resistance of these cancers to apoptotic signals. Further in depth studies in the physio-pathologic role of TRPML-2 channels in normal cells should be required to improve understanding of the molecular mechanisms that regulate their expression and functions in brain tumors.

## MATERIALS AND METHODS

### Cells and tissues

Formalin-fixed paraffin-embedded brain tissues from human tumor biopsies surgically removed from patients who gave informed consent to the study. (n = 63), were kindly provided by Prof. Felice Giangaspero (I.N.M., Neuromed, Pozzilli, Isernia, Italy), Glioma tissues, histologically graded according to the World Health Organization classification criteria [2], were grouped on the basis of malignancy, in pylocitic astrocytomas, grade I (n = 11), astrocytomas, grade II (*n* = 16), anaplastic astrocytomas, grade III (*n* = 17) and GBM, grade IV (*n* = 19).

Breast cancer samples (n = 3) from invasive ER and HER2-positive, high-grade (G3) breast cancers, were collected during surgery and formalin-fixed by the Pathology Unit, AU3, Macerata, from patients giving their informed written consent, that covered the use of their tissues for research purposes. All procedures were conducted in accordance with the Declaration of Helsinki [44, 45].

Messenger RNAs from human normal brain (NB, two different batches NB1 and NB 2) and normal human astrocytes (NHA, two different batches NHA 1 and NHA 2) were purchased from DBA (Milan, Italy). Normal human neural progenitor cells (NS/PCs, two different batches NS/PC 1 and NS/PC 2) were purchased from Cambrex (Berkshire, UK) and maintained in neural progenitor medium (Cambrex). The astrocytoma-glioblastoma U87 (grade III–IV) and glioblastoma T98 and U251 cell lines (grade IV), obtained European Collection of Cell Cultures (ECACC, Salisbury, UK), were maintained in Dulbecco's modified Eagle's medium (DMEM, Lonza Bioresearch, Basel, Switzerland) supplemented with 10% heat inactivated fetal bovine serum (FBS), 2 mmol/L L-glutamine, 100 IU/mL penicillin, 100 μg streptomicin at 37°C, 5% CO_2_ and 95% of humidity. MCF-7 breast adenocarcinoma cell line (ATCC) was cultured in RPMI-1640 medium (Lonza Bioresearch) supplemented with 10% FBS, 100 IU/mL penicillin and 100 μg streptomicin at 37°C, 5% CO_2_ and 95% of humidity.

### Chemical and reagents

3-(4,5-dimethylthiazol- 2-yl)-2,5-diphenyltetrazolium bromide (MTT), bromodeoxyuridine (BrdU), deoxyribonuclease (DNase), propidium iodide (PI), ribonuclease A solution were purchased from Sigma Aldrich (Milan, Italy). Rapamycin was from Adipogen (San Diego, CA, USA). The following rabbit polyclonal antibodies (Abs) were used: anti-ERK (1:1000, Cell Signaling Technology, Denver, CO, USA), anti-caspase-3 (1:1000, Cell Signaling Technology), anti-H2AX (1:1000, Cell Signaling Technology), anti-microtubule-associated protein-1 light chain 3 (LC3, 2 μg/ml, Novus Biologicals, Littleton, CO, USA), anti-p62 (1 : 1000, Cell Signaling Technology), anti-pAKT (1:200, Santa Cruz Biotechnology, Santa Cruz, CA, USA) and anti-mucolipin-2 (anti-MCOLN-2 or anti-TRPML-2, 1:3000 in western blot, 1:500 in immunohistochemistry, Tema Ricerca, Castenaso, Italy). The following mouse monoclonal Abs were used: anti-AKT (1:200, Santa Cruz Biotechnology), anti-pERK (1:2000, Cell signaling Technology), anti-glyceraldehyde-3-phosphate dehydrogenase (anti-GAPDH, 1:3000, Tema Ricerca), mouse IgG1 κ Iso Control (Prodotti Gianni, Milan, Italy) and anti-BrdU fluorescein isothiocyanate (FITC)-conjugated (Prodotti Gianni). The following secondary antibodies were used: horseradish peroxidase (HRP)-conjugated anti-mouse IgG and HRP-conjugated anti-rabbit IgG (GE Healthcare, Munich, Germany), biotinylated anti-rabbit IgG (Bethyl, Montgomery, TX, USA), FITC-conjugated goat anti-rabbit Ab (BD Biosciences, Milan, Italy).

### Western blot

Total lysates from T98, U251, U87 and MCF-7 cell lines were lysed in a lysis-buffer containing protease inhibitor cocktail (Sigma Aldrich). Plasma membrane and cytosol fractions from glioma cell lines were isolated using the Subcellular Protein Fractionation kit (Thermo Scientific, Rockford, IL, USA), according to the manufacturer's directions.

Proteins were separated on 8-14% SDS polyacrylamide gel, transferred onto Hybond-C extra membranes (GE Healthcare) and blotted with the specific Abs. Non-specific binding sites were blocked with 5% low-fat dry milk and 2% bovine serum albumin (BSA) in phosphate-buffered saline 0.1% Tween 20 for 1 h at room temperature. Blots were incubated with the anti-TRPML-2 primary Ab for 25 min at 37°C followed by HRP-conjugated anti-rabbit Ab for 1h at room temperature. Membrane were incubated overnight at 4°C in primary Abs (anti-caspase 3; anti-H2AX, anti-pAKT, anti-AKT, anti-pERK, anti-ERK, anti-p62, anti-LC3, anti-GAPDH), followed by the incubation for 1 h at room temperature with HRP-conjugated anti-rabbit or anti-mouse secondary Abs. The detection was performed using the LiteAblot PLUS kit (EuroClone, Milan, Italy), and densitometric analysis was carried out by a Chemidoc using the Quantity One software (Bio-Rad, Hercules, CA, USA). For quantification, GAPDH was used as loading control. One representative out of three independent experiments is shown.

### TRPML-2 gene silencing

siGENOME SMARTpools for TRPML-2 (siTRPML-2), siCONTROL non-targeting siRNA (siGLO) used as negative control were purchased from Thermo Scientific-Dharmacon (Lafayette, CO, USA). For gene silencing experiments, T98 and U251 cell lines were plated at the density of 3 × 10^4^/ml and 160 pmol of siTRPML-2 or siGLO was added to the wells, following the METAFECTENE SI PRO transfection protocol (Biontex Laboratories, San Diego, CA, USA).

### Cell viability

Cell viability was assessed by the MTT assay. Three x 10^4^ untransfected, siTRPML-2 and siGLO cells/ml were seeded in 96-well plates. After 72 h of incubation, 0.8 mg/ml of MTT was added to the samples and incubated for 3 h. After the removal of medium from the wells, the formazan crystals were dissolved with 100 μl per well of DMSO and the coloured solutions were read by microtiter plate spectrophometer (BioTek Instruments, Winooski, VT, USA). Four replicates were used for each treatment.

### Cell cycle analysis

Cell cycle analysis was performed in untransfected, siTRPML-2 and siGLO cells. After 72 h of transfection cells were fixed in ice-cold 70% ethanol, treated for 30 min at 37°C with 100 μg/ml ribonuclease A solution, stained for 30 min at room temperature with PI 20 μg/ml and analyzed by flow cytometry using linear amplification.

### BrdU cell proliferation assay

BrdU incorporation was determined in untransfected, siTRPML-2 and siGLO cells after 72 h of transfection. Cells were labelled by adding 20 μM BrdU for 24 h and fixed in cold ethanol 70%. After this, cells were washed with PBS and incubated with HCl 2N for 30 min at room temperature. Thereafter, cells were washed twice with PBS and incubated with the anti-BrdU FITC-conjugated Ab, diluted 1:50 in a PBS-T solution (1% BSA and 0.5% Tween 20), for 1 h, in the dark, under agitation. Finally, cells were washed once with PBS-T and incubated with the same solution used for cell cycle analysis, followed by flow cytometry.

### Cell death analysis

Cell death was evaluated using FITC-conjugated Annexin V (Ann V) and PI staining followed by single and biparametric FACS analysis. Briefly, untreated, siTRPML-2 and siGLO cells were incubated with 5 μl Annexin V-FITC (Enzo Life Sciences, Farmingdale, NY, USA) and 20 μg/ml PI for 10 min at room temperature. The cells were then analyzed by flow cytometry using CellQuest software.

### Mitochondrial transmembrane potential (ΔΨm)

Mitochondrial transmembrane potential was evaluated by JC-I staining in untransfected, siTRPML-2 and siGLO cells. After 72 h of transfection cells were incubated for l0 min at room temperature with JC-1. JC-I was excited by an argon laser (488 nm) and green (530 nrn)/red (>570 nrn) emission fluorescence was collected simultaneously. Carbonyl cyanide chlorophenylhydrazone protonophore, a mitochondrial uncoupler that collapses ΔΨm was used as positive control (data not shown). Samples were analyzed by a FACScan cytofluorimeter using the CellQuest software; fluorescence intensity was expressed in arbitrary units on logarithmic scale.

### RNA extraction and reverse transcription

Total RNA from fixed paraffin-embedded tissue slices (5-7 μm-thick) was extracted by RNeasy® FFPE Mini Kit (Qiagen, Milan, Italy). RNA from U87, U251, T98 and MCF-7 cell lines was extracted by RNeasy Mini Kit (Qiagen). All RNA samples were eluted in the appropriate buffer and their concentration and purity were evaluated by A260/280 nm measurement. Five hundred nanograms of RNA extracted were subjected to reverse transcription in a total volume of 25 μl using the high-capacity cDNA archive kit (PE Applied Biosystems, Foster City, CA, USA) according to the manufacturer's instructions. Five microlitres of the cDNA derived by fixed paraffin-embedded tissue was pre-amplified for 15 cycles using RT^2^ PreAMP cDNA Synthesis kit (Qiagen). Two microliters of the resulting cDNA products were used as template for polymerase chain reaction (PCR).

### Quantitative real time polymerase chain reaction (qRT-PCR)

Quantitative RT-PCR was performed by using the IQ5 Multicolor realtime PCR detection system (BioRad, Hercules, CA, USA). The reaction mixture contained the RT^2^ SYBR® Green qPCR Mastermix (Qiagen). Human TRPML-2 primers sequence (forward 5′-CGGCAGCCTTATCGTTTTCC-3′; reverse 5′-GCCATTGCATTTCTGACGGT-3′), GAPDH primers sequence (forward 5′-AGAAAAACCTGCCAAATATGATGAC-3′; reverse 5′-TGGGTGTCGCTGTTGAAGTC-3′;) were designed by Primer Express Software (PE Applied Biosystems) and purchased from Sigma Genosys (The Woodlands, TX, USA). The PCR parameters were 10 min at 95°C followed by 40 cycles of 95°C for 15 s and 60°C for 40 s. All samples were assayed in triplicate in the same plate. The relative amount of target mRNA was calculated by the 2^−ΔΔCt^ method, using GAPDH as a housekeeping gene.

### Immunohistochemistry

For immunohistochemistry, after re-hydration, sections were incubated with Tris-HCl 20 mM, EDTA 0,65 mM, Tween-20 0,0005% pH 9, in a microwave for 5 min (two times) for antigen retrieval. Sections were treated with H_2_O_2_ for 20 min, washed, incubated for 1 h at room temperature with 5% bovine serum albumin (BSA) and 0.3% Triton X-100 in PBS, and then overnight at 4°C with anti-TRPML-2 Ab. Thereafter, slides were incubated for 30 min at room temperature with a biotinylated anti-rabbit secondary antibody (1:200) rinsed, and exposed for 30 min to the streptavidin–biotin complex (VECTASTAIN ABC Kit, Vector laboratories, Burlingame, CA, USA). Immunoreactivity was detected by the addition of diaminobenzidine (Peroxidase Substrate Kit, Vector laboratories). Four random fields of each tissue specimen were analyzed under x20 magnification using the Leica DMR Microscope collected by TV camera with an IAS 2000 system (Delta Sistemi, Rome, Italy). The density of immunoreaction was measured by image analysis in different sample portion. The intensity of immunostaining was evaluated microdensitometrically calibrating the image analyzer, taking as “zero” the background developed in sections incubated with a non-immune serum and “250” as the conventional value of maximum intensity of staining.

U87, U251, T98 and MCF-7 cell lines were maintained on cover slips of 6-well plates in fresh medium. After 24 h cells on cover slips were fixed with 4% paraformaldehyde for 15 min at room temperature, and were then counterstained with haematoxylin. Immunohistochemistry in the cells was performed as above described, without antigen-retrieval.

### Immunofluorescence and FACS analysis

The membranous and intracellular TRPML-2 expression was determined by flow cytometry. Cells were fixed with 4% paraformaldehyde and then each sample was divided into two groups, one to be stained with anti-TRPML-2 Ab (1:100) in staining solution (phosphate-buffered saline [PBS], 1% FBS and 0.1% NaN_3_) and one to be stained with anti-TRPML-2 Ab (1:100) in permeabilization buffer (PBS, 1% FBS, 0.1% NaN3 and 1% saponin). After an incubation of 1 h at 4°C, cells were then incubated with FITC-conjugated anti-rabbit Ab and analysed using a FACScan cytofluorimeter with CellQuest software.

### Statistical analysis

The statistical significance was determined by Student's t-test and by Anova with Bonferroni's post-test. No statistically significant difference was found between untransfected and siGLO transfected U251 and T98 cells (data not shown).

## SUPPLEMENTARY FIGURE


