# Supplementary material for: Overexpression of transient receptor potential mucolipin-2 ion channels in gliomas: role in tumor growth and progression
Source: Oncotarget. 2016 May 27;7(28):43654–68. doi: 10.18632/oncotarget.9661 (PMC5190050; doi:10.18632/oncotarget.9661)
Supplement: Supplementary file 1 [file oncotarget-07-43654-s001.pdf]

Overexpression of transient receptor potential mucolipin-2 ion channels in gliomas: role in tumor growth and progression

SUPPLEMENTARY FIGURE

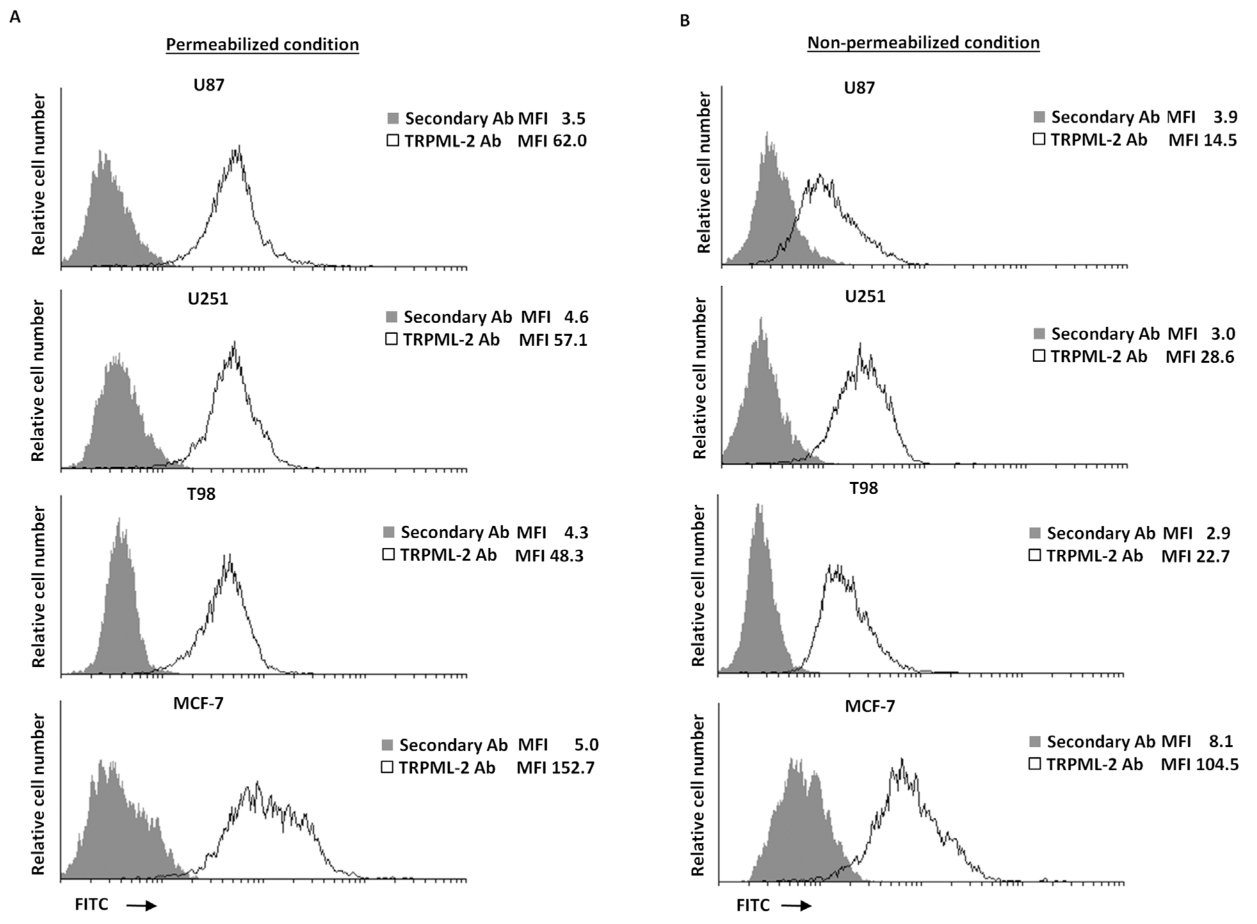

Supplementary Figure S1: TRPML-2 expression and localization in glioma cell lines. Cytofluorimetric analysis of TRPML-2 protein expression in permeabilized **A.** or not permeabilized **B.** U87, T98, U251 and MCF-7 cell lines using specific anti-TRPML-2 Ab. MFI= Mean Fluorescence Intensity
